# TCF-Trans: Temporal Context Fusion Transformer for Anomaly Detection in Time Series

**DOI:** 10.3390/s23208508

**Published:** 2023-10-17

**Authors:** Xinggan Peng, Hanhui Li, Yuxuan Lin, Yongming Chen, Peng Fan, Zhiping Lin

**Affiliations:** 1 School of Electrical and Electronic Engineering, Nanyang Technological University, Singapore 639798, Singapore; xinggan001@e.ntu.edu.sg (X.P.); liny0108@e.ntu.edu.sg (Y.L.); yongming001@e.ntu.edu.sg (Y.C.); 2 School of Intelligent Systems Engineering, Shenzhen Campus of Sun Yat-sen University, Shenzhen 518107, China; lihh77@mail.sysu.edu.cn; 3 Chongqing Yuxin Road & Bridge Development Co., Ltd., Chongqing 400060, China; fanpeng@cftecgroup.com

**Keywords:** anomaly detection, deep learning networks, transformer, time series

## Abstract

Anomaly detection tasks involving time-series signal processing have been important research topics for decades. In many real-world anomaly detection applications, no specific distributions fit the data, and the characteristics of anomalies are different. Under these circumstances, the detection algorithm requires excellent learning ability of the data features. Transformers, which apply the self-attention mechanism, have shown outstanding performances in modelling long-range dependencies. Although Transformer based models have good prediction performance, they may be influenced by noise and ignore some unusual details, which are significant for anomaly detection. In this paper, a novel temporal context fusion framework: Temporal Context Fusion Transformer (TCF-Trans), is proposed for anomaly detection tasks with applications to time series. The original feature transmitting structure in the decoder of Informer is replaced with the proposed feature fusion decoder to fully utilise the features extracted from shallow and deep decoder layers. This strategy prevents the decoder from missing unusual anomaly details while maintaining robustness from noises inside the data. Besides, we propose the temporal context fusion module to adaptively fuse the generated auxiliary predictions. Extensive experiments on public and collected transportation datasets validate that the proposed framework is effective for anomaly detection in time series. Additionally, the ablation study and a series of parameter sensitivity experiments show that the proposed method maintains high performance under various experimental settings.

## 1. Introduction

Anomaly detection aims to find patterns that do not comply with expected behaviour [1]. In many real-world applications, anomaly detection tasks are important research topics [2,3]. Typically, anomalies are categorised as point, contextual, and collective. Since it is common to find that no specific distributions fit the data, and the characteristics of anomalies are different, using traditional anomaly detection methods based on distance estimation or statistical theory may be challenging. Moreover, complex and changing intrinsic data characteristics, low recall rate, and high dimensional data [4] further impede the learning performance of traditional machine learning methods. Under these circumstances, the detection algorithm requires excellent learning ability of the data features. Deep learning methods commonly learn the complex dynamics in the data without relying upon underlying patterns within the data. This advantage makes them popular in dealing with anomaly detection tasks. Transformers, which apply the self-attention mechanism, have shown outstanding performances in modelling long-range dependencies among different deep learning methods.

Common methods for dealing with anomaly detection tasks [5,6,7] can be generally classified as traditional machine learning methods and deep learning methods.

SVM [8], One-class SVM (OC-SVM) [9], Isolation forest [10] and Local Outlier Factor (LOF) [11] are typical examples of machine learning anomaly detection algorithms. However, if raw samples are complex or dense, the detection performance of these methods will be limited. LODA [12] is a lightweight anomaly detector that ensembles different detectors and is suitable for data streams. LSCP [13] is another ensemble framework compatible with different types of base detectors and further determines the most competent base detector in the local region upon similarity measuring.

Although machine learning methods are suitable for some anomaly detection tasks, deep learning methods more effectively learn expressive representations of complex data in some real-world applications [4,14]. For example, deep support vector data description (DeepSVDD) [15] is applied to complex data for better feature selection. Recurrent neural networks (RNN) that capture time dependence are commonly used to recognise or predict sequences. RNN has been exploited with gating mechanisms to become common methods such as LSTM and Gated recurrent units (GRU). For example, one LSTM-based method is adopted for detecting urban anomalies [16]. DeepAnT [17] is a novel anomaly detection method in time series, which does not require a huge dataset. It primarily applies a CNN-based network to take a window range of time series and try to predict its value for the next stamp. Then, the predicted value is sent to an anomaly detector module to determine its abnormality. DAGMM [18] applies a compression network and an estimation network to achieve unsupervised anomaly detection. The compression network implements a deep autoencoder to generate a low-dimensional representation for each input. Then the estimation network, based on the Gaussian Mixture Model, takes the representation and predicts the corresponding likelihood. Parameters of both the two sub-networks are jointly optimised simultaneously. SO-GAAL [19] applies the generative adversarial learning framework, which consists of a generator and a discriminator used to detect anomalies. Alternatively, GDN [20] combines graph structure learning and attention weights to achieve good anomaly detection results in some fields. LUNAR [21] is another graph neural network-based anomaly detection method. It extracts information from the nearest neighbours of each node and further detects anomalies, and it can learn and adapt to different sets of data. Transformer [22]-based algorithms are also widely applied in anomaly detection tasks. For example, UTRAD [23] obtains stable training and accurate anomaly detection/localisation results based on a transformer-based autoencoder. Additionally, MT-RVAE [24] utilises the variational Transformer model with improved positional encoding and feature extraction to achieve satisfying anomaly detection performances.

**Figure 1:**
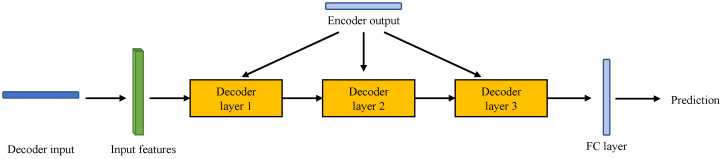
Demonstration example of the 3-layer decoder block diagram for the original Informer network.

In this paper, one Transformer-based network, Informer [25], is chosen as the baseline for dealing with anomaly detection tasks with data collected from real-world applications. The original Informer is an efficient model of Transformer that adopts *ProbSparse* self-attention mechanism to significantly reduce the time complexity and memory usage while outperforming existing methods, mainly in time-series forecasting tasks. Additionally, it can handle tasks in an unsupervised way to avoid the cumbersome labelling cost. However, directly applying the original Informer to time-series anomaly detection tasks may not be appropriate. Since the origin Informer is used in time-series forecasting tasks, it aims to find the overall trend of the target sequence and can ignore some unusual details. Moreover, Transformers may focus on dominant relationships among sequences while paying less attention to intrinsic details when dealing with short-term data. As a result, as shown in Figure 1, it has a straight-throughout feature-transmitting structure for layers in the decoder, and the output of the informer decoder is merely based on the last layer, which contains the least noise but may miss some details. However, anomalies may be rare in anomaly detection tasks, and some minor details in data may reflect the anomaly and cannot be ignored. Different features should be utilised to improve the overall detection performances [26].

To overcome the above-mentioned limitations, we propose to better utilise features from shallow and deep decoder layers with a new multi-layer feature fusion decoder. The original feature-transmitting structure in the decoder of Informer is replaced with the proposed feature fusion decoder to fully utilise the features extracted from shallow and deep decoder layers. This strategy prevents the decoder from missing unusual anomaly details while maintaining robustness from noises inside the data. Next, the auxiliary predictions generated by the decoder will be further adaptively fused based on similarities/distances and sequence information in the temporal context fusion module. This strategy exploits temporal context information of the data by a learnable weight to make the output more robust. We evaluate the proposed method using both the public and our collected transportation datasets for anomaly detection tasks and compare the results with recently proposed machine learning and deep learning methods.

The main contributions of our work can be summarised as follows:

- We introduce a novel framework: Temporal Context Fusion Transformer (TCF-Trans) for unsupervised anomaly detection in time series based on temporal context fusion.
- We replace the straight throughout feature-transmitting structure in the decoder layers of Informer with the proposed feature fusion decoder, which fully utilises the features extracted from shallow and deep decoder layers. This strategy prevents the decoder from missing unusual anomaly details while maintaining robustness from noises inside the data.
- We propose the temporal context fusion module to fuse the auxiliary predictions generated by the decoder adaptively. This strategy alleviates noises or distortions caused by the single auxiliary prediction and fully uses temporal context information of the data.
- Extensive experiments on the public and collected transportation datasets validate that the proposed framework is effective for anomaly detection tasks, such as transportation tasks in time series. In addition, a series of sensitivity experiments and the ablation study show that the proposed method maintains high performance under various experimental settings.

The remaining parts of this paper are organised as follows. Section 2 reviews related works and the background of the Transformer. Section 3 describes the details of the proposed method. Section 4 describes the experiments for validating the proposed method. The conclusion of this paper is presented in Section 5.

## 2. Related Work

In this section, the background and a review of related works are presented. We first formulate the anomaly detection task in Section 2.1. The background of the transformers and Informer are described in Section 2.2 and Section 2.3, respectively.

### 2.1. Problem Statement

Given a time-series x$\in R^{C\times L}$, where *C* is the number of channels, and *L* is the length of the sequence, we can obtain an observation sequence $X^{t}$ based on x. The observation at time *t* with length $L_{x}$ can be represented as follows:

(1) $$\begin{array}{cc} \; & X^{t}=\left\{x_{1},\ldots ,x_{L_{x}}\;|\;x_{i}\in R^{d_{x}}\right\}, \end{array}$$

where $x_{i}$ is the *i*th observed values and $d_{x}$ is the dimension of the observation.

Then, we follow the forecasting-based strategy to generate the corresponding prediction sequence $Y^{t}$ with length $L_{y}$ based on $X^{t}$, as follows:

(2) $$\begin{array}{cc} \; & {\hat{Y}}^{t}=\left\{{\hat{y}}_{1},\ldots ,{\hat{y}}_{L_{y}}\;|\;{\hat{y}}_{i}\in R^{d_{y}}\right\}, \end{array}$$

where ${\hat{y}}_{i}$ is the *i*th predicted value and $d_{y}$ is the dimension of the prediction.

Time series anomaly detection methods aim to determine anomalies in x. Our anomaly detection approach is to generate an anomaly score based on anomaly criteria between $\hat{Y}$ and its corresponding target Y. Next, the anomaly detection result can be obtained by comparing the anomaly score to a threshold.

### 2.2. Transformer

Compared with traditional machine learning methods, deep learning methods can capture complex dynamics in the data without making assumptions about the raw data. Among numerous deep learning methods, LSTM [27] and Transformer [22] based algorithms are popular in time series tasks, including anomaly detection. Although LSTM-based methods achieve satisfying performances in some anomaly detection tasks, their highly computationally expensive and inefficient long temporal pattern learning capability still limits their performances. Recently, innovations based on the vanilla Transformer [22] have been widely applied in various fields such as natural language processing (NLP) [28,29], computer vision (CV) [30,31,32,33] and time series applications [34,35,36].

**Figure 2:**
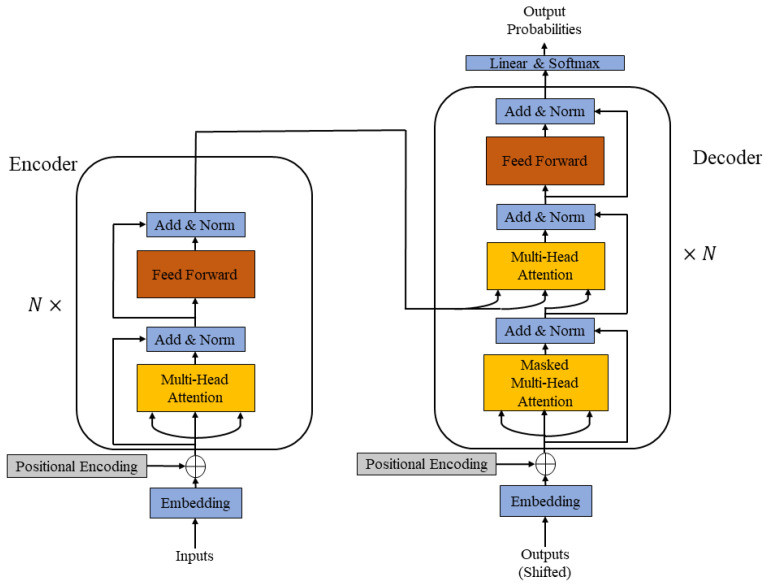
Architecture of vanilla Transformer.

Transformers have shown outstanding performances in modelling long-range dependencies based on the self-attention mechanism and therefore are appealing for time series applications. Moreover, compared with LSTM-based methods, Transformer-based methods are commonly more efficient because of parallel computing [37]. The architecture of a vanilla Transformer framework is shown in Figure 2. The vanilla Transformer consists of an encoder and a decoder, each of which is a stack of *N* identical layers. Each encoder layer contains two sub-layers, including the multi-head self-attention module and a position-wise fully connected feed-forward network. The residual connection [38] is applied on each sub-layer, and a layer normalisation module [39] is set at the end of each sub-layer. As for the decoder, it has one additional layer between the two sub-layers to perform multi-head attention over the output of the encoder. The residual connection and layer normalisation modules are also implemented in the decoder. Alternatively, a mask is added to the self-attention module to prevent positions from attending to subsequent positions.

The vanilla Transformer has four key modules: the attention module, feed-forward module, layer normalisation module, and positional encoding module [40].

The attention module takes the scaled dot-product attention with Query-Key-Value (QKV) model, which is shown in Equation (3).

(3) $$\mathrm{Attention}\left(Q,K,V\right)=\mathrm{softmax}\left(\frac{QK^{T}}{\sqrt{D_{k}}}\right)V$$

where packed matrix representations of queries $Q\in R^{L_{Q}\times D_{k}}$, keys $K\in R^{L_{K}\times D_{k}}$, values $V\in R^{L_{k}\times D_{V}}$ and $L_{Q}$, $L_{K}$ are lengths of queries and keys (or values). $D_{k}$, $D_{V}$ are dimensions of keys (or queries) and values, respectively.

Then, the vanilla Transformer applies the multi-head attention to project the queries, keys and values with *H* different sets of learned projections, which is shown in Equation (4).

(4) $$\mathrm{MultiHeadAttn}\left(Q,K,V\right)=\mathrm{Concat}\left(\mathrm{head}{}_{1},\ldots ,\mathrm{head}{}_{H}\right)W^{O}$$

(5) $$\mathrm{where}\;\mathrm{head}{}_{i}=\mathrm{Attention}(QW_{i}^{Q},KW_{i}^{K},VW_{i}^{V})$$

where $W_{i}^{Q}$, $W_{i}^{K}$, $W_{i}^{V}$ and $W^{O}$ are projection parameter matrices.

The vanilla Transformer performs remarkably in many fields, but quadratic computation complexity remains the bottleneck for some real-world tasks. Recently, Informer [25] selects prototypes from queries using *ProbSparse* mechanism based on Kullback–Leibler divergence between the query’s attention probability distribution and the uniform distribution. However, as we mentioned earlier, directly applying the Informer to anomaly detection has limitations. Therefore, a novel temporal context fusion framework named TCF-Trans based on Informer [25] is presented in the next section to better deal with anomaly detection tasks in time series.

### 2.3. Preliminary on Informer

Although the vanilla Transformer performs remarkably well in many fields, high computational complexity remains challenging for some real-world tasks. Several works have been proposed to improve the efficiency of the vanilla Transformer. Informer [25] is one of the most popular improved versions. It selects prototypes from queries using *ProbSparse* mechanism based on the Kullback–Leibler divergence between the query’s attention probability distribution and the uniform distribution. Informer significantly reduces the time complexity and the whole memory usage of the network while maintaining the good learning capability of the Transformer.

The Informer [25] implements a popular encoder-decoder architecture to produce outputs. The encoder encodes the input $X^{t}$ into the hidden state representation $H^{t}$. Next, the decoder produces output ${\hat{Y}}^{t}$ based on $H^{t}$.

The standard self-attention can be replaced by the *ProbSparse* self-attention mechanism to reduce the time complexity from $O(L^{2})$ to O(LlnL). This mechanism allows each key to only attend to the Top-*k* dominant queries, as follows:

(6) $$\begin{array}{c} A\left(Q,K,V\right)=\mathrm{Softmax}\left(\frac{\bar{Q}K^{T}}{\sqrt{d}}\right)V, \end{array}$$

where $\bar{Q}$ is the sparse matrix of queries that only contains top-*k* dominant of queries under the query sparsity measurement [25], *d* is the dimension of the input, K is the matrix of keys and V is the matrix of values.

In the encoder, Self-attention distilling operations can also be implemented between attention blocks to reduce the whole memory usage. The encoded feature will be sent to the decoder layers to produce auxiliary predictions.

As shown in Figure 1, the structure of the Informer decoder contains layer stacks of multi-head attention blocks. The decoder input combines the earlier piece sequence before the output and a placeholder, as follows:

(7) $$\begin{array}{cc} \; & X_{din}^{t}=\left[X_{ref}^{t},X_{0}^{t}\right]\in R^{\left(L_{ref}+L_{y}\right)\times d_{model}}, \end{array}$$

where [·,·] donates the concatenation, $X_{ref}^{t}$ is a $L_{ref}$ long reference sequence, $X_{0}^{t}$ is a $L_{y}$ long placeholder sequence and $d_{model}$ is the dimension of the model.

When computing *ProbSparse* self-attention in the decoder, a mask can be applied by setting masked dot products to −∞ for inappropriate connections. The final output is produced by a fully connected (FC) layer. The decoder of the Informer utilises multiple layers to extract features and reduce noise, which may be beneficial in forecasting applications, since its goal is to determine the overall trends, and hence minor details may not be necessary. However, since anomalies are rare and some points anomalies differ from the primary trend, this setting becomes inappropriate in anomaly detection tasks. Moreover, different features contain different characteristics, and relying on a single feature may make the method less robust and more likely to be infected by noise in the single feature.

## 3. TCF-Trans: Temporal Context Fusion Transformer

### 3.1. Overall Structure

**Figure 3:**
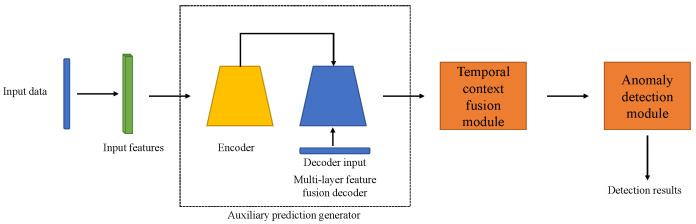
Block diagram of overall structure of TCF-Trans.

As shown in Figure 3, TCF-Trans consists of three main modules: an auxiliary prediction generator, a temporal context fusion module and an anomaly detection module. The auxiliary prediction generator performs feature learning, fusion and refinement of the processed input data based on an Encoder–decoder architecture. Next, the generated auxiliary predictions are further processed by the temporal context fusion module based on the similarity/distance and sequence information to generate output predictions adaptively. Finally, the output predictions are compared with the target data under anomaly detection criteria to produce anomaly scores. The final detection result will be determined based on the threshold. These modules will be presented in detail in the following sections.

### 3.2. Auxiliary Prediction Generator

The auxiliary prediction generator implements an Encoder–decoder architecture similar to the Informer [25]. The input $X^{t}$ is encoded into the hidden state representation $H^{t}$ as the encoder output. Next, the decoder produces output auxiliary predictions ${\hat{P}}^{t}$ based on the encoder output $H^{t}$ and the decoder input $X_{din}^{t}$.

**Figure 4:**
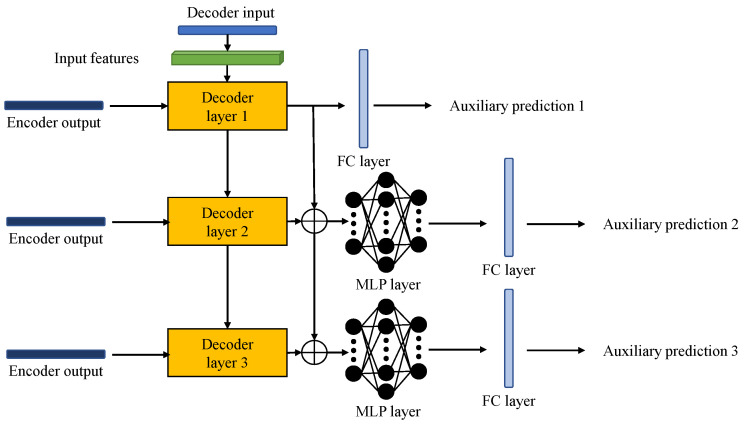
Demonstration example of the three-layer feature fusion decoder of TCF-Trans. ⊕ donates feature fusion operations.

The process in the encoder is based on the Informer baseline. However, as we mentioned earlier, the process of the Informer baseline in the decoder is ineffective in anomaly detection tasks. To overcome such limitations, we propose a multi-layer feature fusion decoder to better use different features extracted in shallow and deep decoder layers and further refine them to generate auxiliary predictions. To smoothly demonstrate the idea of feature fusion in the decoder, we take a three-layer decoder as an example. As shown in Figure 4, the decoder consists of three layers and inputs are based on encoder output $H^{t}$ and the decoder input $X_{din}^{t}$. The outputs for three layers are denoted as $D=\{d_{1},d_{2},d_{3}\}$, where $d_{i}$ is the output for the *i*th layer. Our feature fusion aims to generate the merge and refined $d_{i}\in R^{L}$ (denoted as $d_{i}^{*}$) for the *i*th layer based on D and use it to produce auxiliary predictions to assist detection.

In the multi-layer decoder, shallow layers contain more information about details, while deep layers contain more depth representations of the data [41,42]. Therefore, we can fuse features from deep layers with those from shallow layers to obtain an optimal representation of the data, as follows:

(8) $$\begin{array}{cc} \; & d_{i}^{*}=\left[d_{i},\ldots ,d_{1}\right]\in R^{L\times d_{feature^{*}}}, \end{array}$$

where [·,…,·] donates the concatenation, *i* is the order of the layer and $d_{feature^{*}}$ is the dimension of the fused feature.

During the fusion process, the deeper the layer, the more features it fuses. Under this circumstance, directly applying the FC layer to produce outputs makes it likely to lose information. Therefore, feature-refining tools such as multilayer perceptron (MLP) layers can be implemented to refine the fused features for deeper layers. Note that MLP layers can be represented as hidden layers in the MLP network and layer normalisation can be added to achieve stable transmission.

Lastly, auxiliary predictions produced by the FC layer using refined features can be defined as follows:

(9) $$\begin{array}{cc} \; & {\hat{P}}^{t}=\left\{{\hat{P}}_{1}^{t},\ldots ,{\hat{P}}_{N}^{t}\right\}, \end{array}$$

where *N* is the number of layers in the decoder and ${\hat{P}}_{1}^{t}$ is the *i*th auxiliary prediction.

During the training of this module, the mean squared error (MSE) loss function can be chosen to compute loss among auxiliary predictions and target sequences. Moreover, weights can be assigned for each prediction to emphasise the importance of predictions produced by different layers. The loss function of this module can be expressed as follows:

(10) $$\begin{array}{ccc} \; & Loss_{1}=\sum_{i=1}^{N}w_{i}MSE({\hat{P}}_{i}^{t}, & Y^{t}), \end{array}$$

where $w_{i}$ is the weight of the *i*th decoder layer.

### 3.3. Temporal Context Fusion Module and Anomaly Detection

After obtaining several auxiliary predictions produced by the former module, one direct way to combine them is to empirically assign weights to each prediction. However, such a method takes a long time to reach a satisfying solution due to the large number of trials required. Also, applying a scalar weight limited the utilisation of the temporal context of these predictions. Because different dimensions or points in the sequence may include different intrinsic temporal contexts or the importance of one prediction, applying a scalar weight to a prediction means only different importance exists on different predictions. However, all points in the sequence and dimensions may not share the same importance. In that case, the output prediction based on a fixed scalar weight may only partially take advantage of our feature fusion decoder. Therefore, we aim to produce the final prediction adaptively based on a weight learned from these auxiliary predictions’ similarity/differences and fused temporal context.

**Figure 5:**
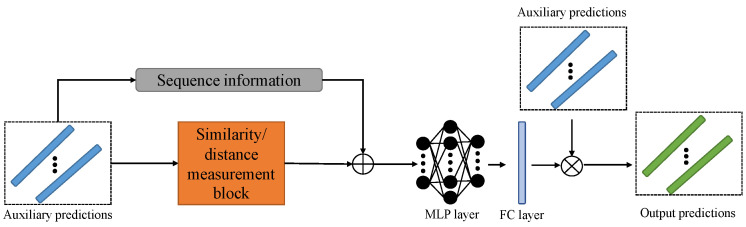
Demonstration example of temporal context fusion module of TCF-Trans. ⊕ donates feature fusion operations.

As shown in Figure 5, auxiliary predictions are sent in the similarity/distance measurement block to determine their similarities/differences. We can calculate the similarities/differences among them as follows:

(11) $$\begin{array}{cc} \; & d_{i,j}=k\left({\hat{P}}_{i}^{t},{\hat{P}}_{j}^{t}\right)\;\;i\neq j, \end{array}$$

where k(·,·) can be a user-defined distance or similarity measurement such as Euclidean distance, etc.

In the meantime, a slice or all of each auxiliary prediction $▵P_{i}^{t}\in R^{L_{s}\times d_{s}}$ can be chosen as the sequence information, where $L_{s}$ and $d_{s}$ are its length and dimension, respectively. Target sequences could also be added to the sequence information, and we take the auxiliary prediction as an example for convenience of understanding. Then, the temporal context fusion is processed as follows:

(12) $$\begin{array}{c} {\hat{D}}^{t}=\left[d_{1,2},\ldots ,d_{N,N-1},▵P_{1}^{t},\ldots ,▵P_{N}^{t}\right], \end{array}$$

where [·,…,·,·,…,·] donates the concatenation and *N* is the number of layers in the decoder.

Next, the fused temporal context is processed by MLP layers to further feature extraction and refinement. Since we aim to make full use of auxiliary predictions’ similarity/differences and temporal information, the output of the MLP will be sent to the FC layer to adaptively generate the weight $W^{t*}$ based on different weight resolution expectations on various data inputs. (learned weight with a higher dimension presents higher resolution). Specifically, the learned weight $W^{t*}$ can be chosen from $W^{t}=\left\{W^{t}\in R^{N},W^{t}\in R^{d_{y}\times N},W^{t}\in R^{L_{y}\times d_{y}\times N}\right\}$.

Last, the output prediction can be computed as follows:

(13) $$\begin{array}{c} {\hat{Y}}^{t}=\sum_{i=1}^{N}W_{i}^{t*}\cdot {\hat{P}}_{i}^{t}. \end{array}$$

During the training of this module, we can also implement the MSE loss function to compute loss output prediction and target sequences.

After we have obtained the output predictions, the anomaly detection module compares them with the target sequence under specific criteria. Here, we can choose MSE loss to generate the anomaly score as follows:

(14) $$\begin{array}{cc} \; & AnomalyScore=MSE(Y,\hat{Y}). \end{array}$$

Once we have the anomaly score, a threshold can be set to determine anomalies. The threshold can be determined empirically or via grid search for better precision, but such methods require extensive trials and are time-consuming. Alternatively, methods based on Streaming Peaks-Over-Threshold (SPOT) [43] can be applied to determine the threshold th.

Therefore, values exceeding th in the AnomalyScore are considered potential anomalies. The main procedures for detecting anomalies via TCF-Trans are summarised in Algorithm 1.
**Algorithm 1:** Anomaly detection via TCF-Trans
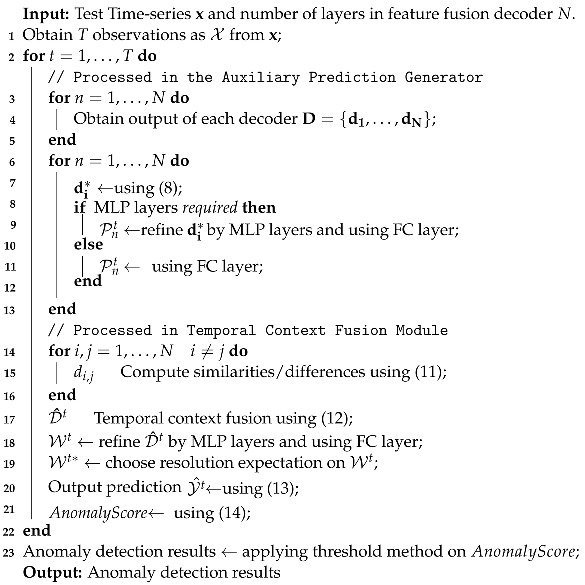


## 4. Experiments

In this section, we validate the effectiveness of the proposed anomaly detection framework through several experiments. First, we describe the experimental setup. Then, we evaluate the proposed framework on three public datasets in Section 4.2. Next, we compare the proposed method with several state-of-the-art methods in the real-world transportation traffic dataset in Section 4.3. Moreover, we conduct an ablation study and parameter sensitivity experiments in Section 4.4 and Section 4.5, respectively.

### 4.1. Setup

We follow standard evaluation metrics in anomaly detection tasks, including $F_{1}$ score, Precision, and Recall, to evaluate performances as follows

(15) $$\begin{array}{cc} \; & Precision=\frac{TP}{FP+TP},\\ \; & Recall=\frac{TP}{FN+TP},\\ \; & F_{1}=2\times \frac{Precision\times Recall}{Precision+Recall}, \end{array}$$

where TP denotes the number of correct anomalous detections, FP denotes the number of incorrect anomalous detections, and FN denotes the number of incorrect normal detection. A higher $F_{1}$ score, precision and recall demonstrate better performances.

The proposed method is implemented with the Pytorch [44] framework and runs on the NVIDIA RTX 3080 GPU. Some comparison methods are based on publicly available codes provided by PyOD [45]. We implement three layers (l=3) for the feature fusion decoder and the dimension of the model $d_{model}=512$.

### 4.2. Evaluation on Public Datasets

We evaluate the proposed method by applying it to three real-world public anomaly detection datasets. The first public dataset is a gesture dataset [46] collected in a real-world scenario. It records *X* and *Y* moving coordinates of one actor’s right hand into a time-series sequence. During the actor’s actions with the right hand, anomalous actions within a specific time period are recorded. In total, the number of data points in the gesture dataset is around 11,000, with a dimension of two. Around 70% of the samples are used to train the model, while the rest of the data are used for testing. The second and the third public datasets are provided in NAB (The Numenta Anomaly Benchmark) [47] with known anomaly causes collected in the real-world scenario. The second one contains temperature sensor data of an internal component of a large industrial machine (i.e., machine temperature dataset). The third one contains ambient temperature data in an office setting (i.e., ambient temperature dataset). Each dataset has more than 7000 univariate data samples collected in time series. Part of the dataset is used as the training set, while the rest is set for testing.

**Figure 6:**
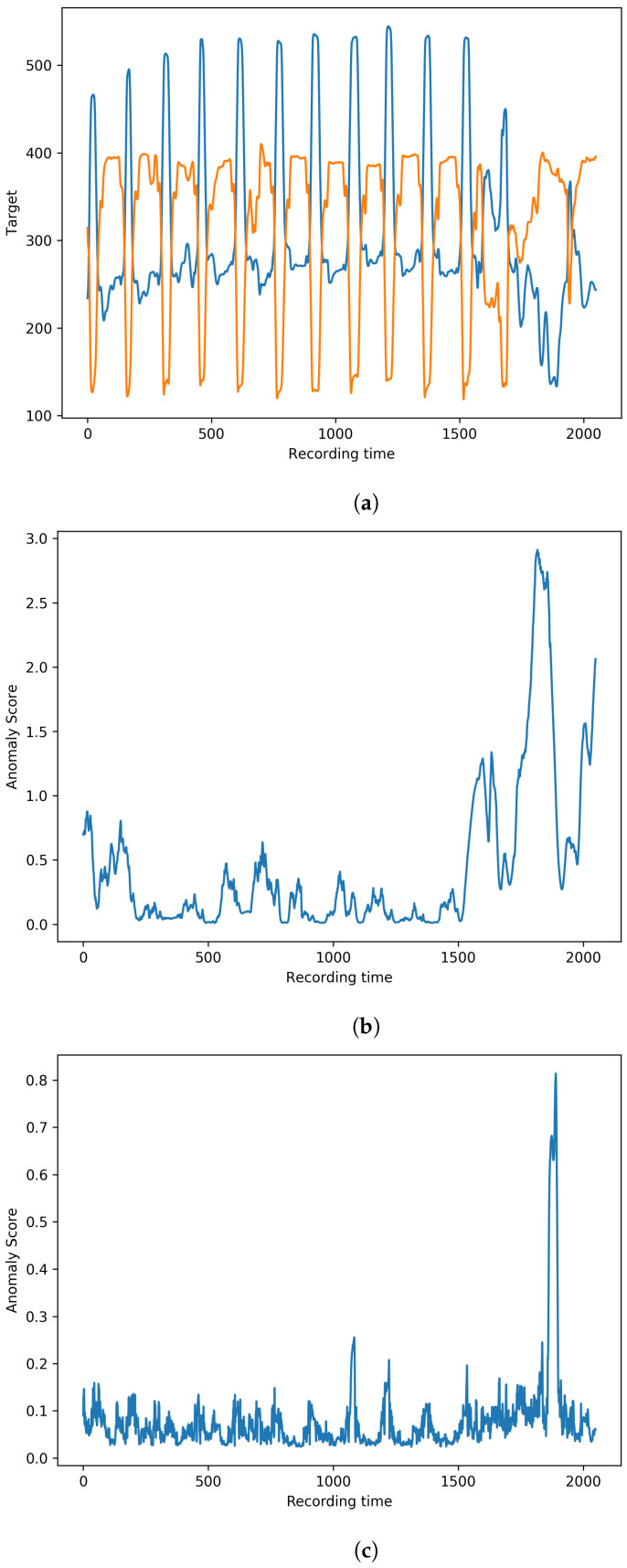
Visualisation examples on the gesture dataset. (**a**) Raw data slice from the test set (**b**) Visualisation example of a detection result slice by the proposed method (**c**) Visualisation example of a detection result slice by LUNAR.

In the gesture dataset, we implement three state-of-the-art comparison methods, including LUNAR [21], DeepAnt [17], and DeepSVVD [15]. Comparison anomaly detection results on this dataset are shown in Table 1, where bold faced number in each column of the table indicates the best result among all the methods in comparison, which is applied to all other tables in this paper. The results show that the proposed TCF-Trans obtains the best performance in terms of $F_{1}$ score, which indicates this method has a good balance in terms of its overall performance without biased detection. Although the recall of the proposed method is not the highest, the methods with higher recall values can suffer from low precision. This phenomenon means they are highly likely to generate false alarms. Therefore, the proposed method obtains competitive performances among the compared state-of-the-art anomaly detection methods. The visualisation examples of detection result slices achieved via the proposed method and LUNAR on the gesture dataset are presented in Figure 6. The X-axis represents recording time. Figure 6a represents the raw data from the test set. Figure 6b and Figure 6c are the corresponding anomaly scores of the proposed method and LUNAR, respectively. Potential anomalies continuously happen after around the 1600th recording time. Compared with LUNAR, the proposed method has more stable anomaly scores among the anomalous regions. On the contrary, anomaly scores obtained from LUNAR suffer from many missing alarms.

In the machine temperature dataset and the ambient temperature dataset, we implement four state-of-the-art comparison methods, including LSCP [13], LUNAR [21], SO-GAAL [19], and DeepSVVD [15]. Comparison anomaly detection results on the machine temperature dataset are shown in Table 2. Results show that the proposed method outperforms other methods in the $F_{1}$ score, with close precision and recall values, indicating that the proposed method can avoid biased detection results. Although some methods have higher recall values than the proposed method, the gap between their recall and precision is noticeable. This unbalanced performance prevents such methods from making accurate predictions among normal samples.

Table 3 summarises the comparison anomaly detection results on the ambient temperature dataset. Results show that TCF-Trans obtains satisfying results among the four methods compared here. Meanwhile, we notice that performance vibrations of some methods on these three datasets are apparent, while the proposed method is more stable for different datasets. This advantage indicates that the proposed method is less sensitive to the change in the intrinsic dataset and may be exploited for many detection tasks.

### 4.3. Evaluation of the Real-World Transportation Dataset

**Figure 7:**
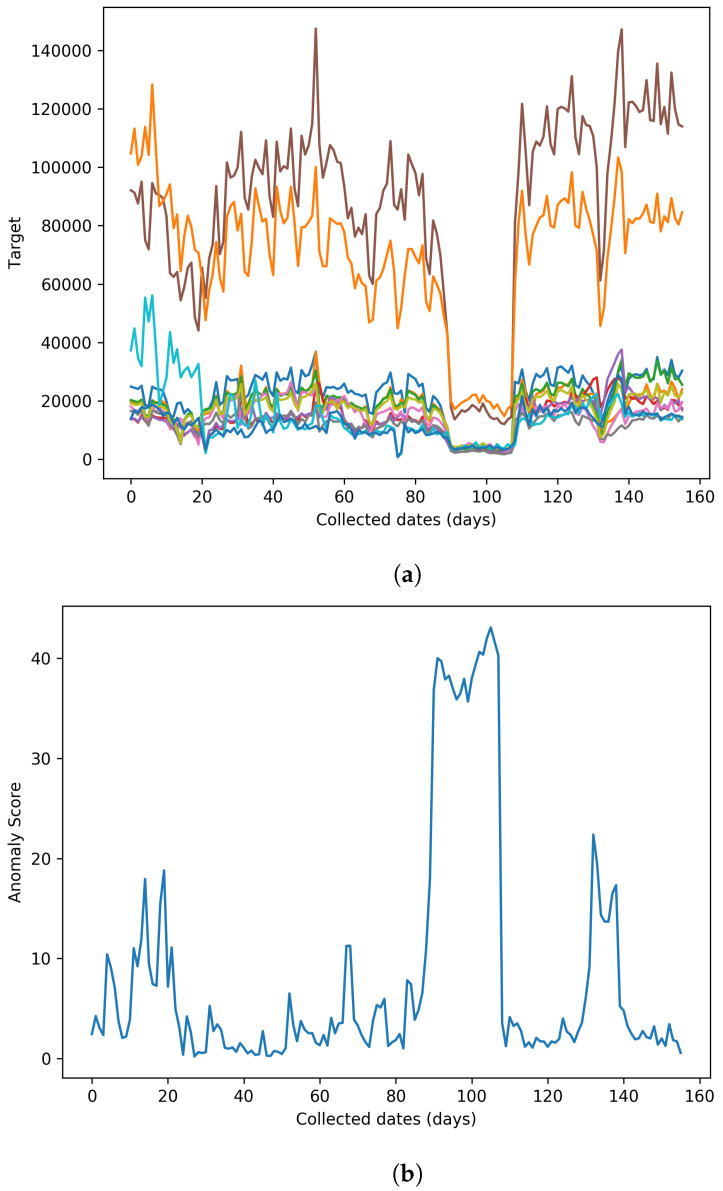
Visualisation examples on the real-world transportation dataset (days). (**a**) Raw data slice from the test set, where each graph line represents traffic from different raw data sources (**b**) visualisation example of a detection result slice achieved using the proposed method.

The proposed method is applied to one real-world collected transportation dataset for anomaly detection tasks to show its potential in other real-life applications. The dataset is collected in Chongqing, China, by days (i.e., real-world transportation dataset (days)), and it contains vehicle traffic data from several roads. This dataset with a day collection rate can reflect long-term traffic anomalies, which can be valuable for local enforcement, helping to assess overall traffic planning and management. Moreover, since the relatively large collection rate requires a longer accumulation of data to enlarge its size, the collection difficulty is greater, and the relatively small size of this dataset already contains information for several months. Under such circumstances, the proposed method’s learning ability with a small number of samples can also be evaluated. The statistical summary of this dataset is shown in Table 4. In total, the real-world transportation dataset (days) contains 270-day vehicle traffic data from different roads. Part of the dataset, which does not contain an anomaly, is used as the training set, while the rest is chosen as the testing set. We implement five state-of-the-art anomaly detection methods for comparison, including LODA [12], LSCP [13], LUNAR [21], SO-GAAL [19], and DeepSVVD [15]. Table 5 presents the comparison anomaly detection results on this dataset. Results show that TCF-Trans effectively detects anomalies in the real-world transportation dataset. It can be observed that the proposed method balances precision and recall. We owe this good ability to the proposed feature fusion strategy because we fuse different features with different characteristics. This strategy can alleviate the negative drawbacks of being vulnerable to noises or missing anomaly details. As a result, the proposed method can obtain good overall detection performance. The visualisation example of a detection result slice is shown in Figure 7. In this figure, the X-axis represents collected dates. Figure 7a contains the raw data slice from the test set, and Figure 7b shows the corresponding anomaly score among the test set. SPOT, which is mentioned in Section 3, can be used to determine the threshold without requiring time-consuming grid search trails. It can be observed that there are two types of anomalies in the raw data. For example, a continuous long-term vehicle traffic drop happens around 100th date, which may reflect a continuous anomalous vehicle traffic drop due to large-scale traffic control by local enforcement. The corresponding anomaly score achieved using the proposed method remains continuously high without a clear drop during this region, which reflects that it can effectively deal with this anomalous pattern. Some short-term sharp changes, such as points before the 140th date, may reflect temporary constructions and belong to another type of anomaly. The anomaly scores in this region are also sufficiently stable, showing that the proposed method can effectively detect short-term anomalies.

### 4.4. Ablation Study

In this section, we conduct the ablation study on the real-world transportation dataset (days) to analyse the effectiveness of each component of the proposed method. We implement four variants of the proposed method, including (i) the Informer baseline, (ii) the TCF-Trans w/o temporal context fusion, in which we replace the temporal context fusion module with one FC layer to generate the output directly, and (iii) the TCF-Trans w/o feature fusion, in which we do not fuse features form different layers. To minimise the impact of different threshold methods, we present results via the grid search based on $F_{1}$ score to check performances in theory (with notations †) in this ablation study.

Based on results shown in Table 6, we make the following observations: (1) The proposed TCF-Trans utilises the advantages of each sub-module to achieve the optimal performance among these variants. (2) Since features from different layers have different characteristics, fusing features from different layers helps to improve detection performances. Fusing them can make the proposed method robust to noise and prevent the decoder from missing potential details related to anomalies. (3) Adaptively fusing the auxiliary predictions based on temporal context helps to satisfactorily generate results. On the contrary, directly transforming auxiliary predictions to the final output may be inappropriate.

Next, we implement another optimiser SGD on the proposed method to evaluate its impacts. Table 7 shows the results of using different optimisers. The results obtained using SGD are worse than the Adam. The low performance can be led by SGD trapping in the local optimal.

Moreover, we gradually decrease the number of data used for training from 100% to 80% to show the proposed method’s potential to achieve effective detection performances without requiring the accumulation of a large amount of data. The results using different ratios of data used are listed in Table 8. It can be found that $F_{1}$ scores do not vary much with ratios from 100% to 80%, which indicates that the proposed method has the potential to obtain satisfying detection performances. Moreover, the results further validate the proposed method’s learning ability with few samples.

### 4.5. Parameter Sensitivity Experiments

In this section, the proposed method is implemented under different parameter settings to evaluate its sensitivity to parameter changes on the real-world transportation dataset (days). We also report results achieved via a grid search, in theory (with notations †), to reduce the impact of different threshold methods.

Since the decoder input $X_{din}^{t}$ combines the earlier piece sequence before the output and a placeholder, we aim to evaluate the effect of reference sequence $X_{ref}^{t}$ with different lengths $L_{ref}$, as well as corresponding input sequence lengths. Therefore, we choose five reference and input length combinations to evaluate their effect. The results of the proposed method using different combinations of lengths are summarised in Table 9. Our $F_{1}$ scores with different lengths are close, which indicates that the proposed method is good at dealing with different input and reference sequences.

Meanwhile, we aim to determine the impact on a larger number of decoder layers. We increase the number of decoder layers to four (i.e., four-layer TCF-Trans) to validate its performance. We also compare the performances of the Informer baseline with four decoder layers (i.e., four-layer Informer baseline). As shown in Table 10, when comparing performances with the four-layer Informer baseline, the impacts of increasing decoder layers for the proposed method are not serious. This can be explained by our fusion strategy, which fully utilises features from shallow and deep layers to make the method more robust to the noise from a single layer while retaining important details for anomaly detection.

Other loss functions besides the MSE loss may also be chosen as the training loss during the training. We also evaluate the proposed method using two different training loss functions: MAE loss and SmoothL1 loss. The results of using different types of training loss are summarised in Table 11. These results show that $F_{1}$ scores with different types of training loss do not vary much, which indicates that loss functions can be adopted in our method.

Based on experiments conducted on the proposed method, the proposed method can handle anomaly detection tasks with different settings, and it shows robustness to these changes. Therefore, the proposed method: TCF-Trans, is an effective solution for anomaly detection in time series applications.

## 5. Conclusions

This paper has addressed anomaly detection tasks in time series based on a novel framework named temporal context fusion transformer (TCF-Trans). This model utilises the Transform’s excellent long-range dependencies modelling capacities and fuses features extracted from shallow and deep decoder layers to prevent the decoder from missing unusual anomaly details while maintaining robustness from noises inside the data, and it improves detection performances. Additionally, the proposed temporal context fusion module fuses the auxiliary predictions generated by the decoder adaptively. It makes the output more robust to noises or distortions caused by the single auxiliary prediction and fully uses temporal context information of the data with a learnable weight. We have performed extensive experiments using the proposed framework on the public and collected transportation datasets. Results have shown that the proposed framework is applicable for some real-world anomaly detection tasks such as transportation in time series. Additionally, the ablation study and several parameter sensitivity experiments have shown that the proposed method can maintain a high performance under various experimental settings.

## Figures and Tables

**Table 1:** Comparison anomaly detection results of the proposed method with other methods on the real-world gesture dataset.

Method	$F_{1}$ (%)	Precision (%)	Recall (%)
LUNAR [21]	50.33	38.57	72.40
DeepAnt [17]	40.08	25.07	**99.86**
DeepSVVD [15]	50.00	43.46	58.86
**TCF-Trans**	**69.69**	**61.67**	80.11

**Table 2:** Comparison anomaly detection results of the proposed method with other methods on the machine temperature dataset.

Method	$F_{1}$ (%)	Precision (%)	Recall (%)
LSCP [13]	60.53	62.30	58.86
LUNAR [21]	45.59	**88.66**	30.69
SO-GAAL [19]	59.76	57.61	**62.08**
DeepSVVD [15]	54.51	81.62	40.92
**TCF-Trans**	**62.55**	68.76	57.36

**Table 3:** Comparison anomaly detection results of the proposed method with other methods on the ambient temperature dataset.

Method	$F_{1}$ (%)	Precision (%)	Recall (%)
LSCP [13]	41.24	48.87	35.67
LUNAR [21]	44.33	35.13	60.06
SO-GAAL [19]	40.36	40.50	40.22
DeepSVVD [15]	32.19	20.41	76.03
**TCF-Trans**	**60.55**	**50.18**	**76.31**

**Table 4:** Statistical summary of the real-world transportation dataset (days).

Dataset	Dimension of Data	Training Size	Testing Size
real-world transportation dataset (days)	12	104	166

**Table 5:** Comparison anomaly detection results of the proposed method with other methods on the real-world transportation dataset (days).

Method	$F_{1}$ (%)	Precision (%)	Recall (%)
LODA [12]	83.05	81.67	84.48
LSCP [13]	90.91	96.15	86.21
LUNAR [21]	88.89	96.00	82.76
SO-GAAL [19]	82.64	79.37	86.21
DeepSVVD [15]	87.39	85.25	89.66
**TCF-Trans**	**93.81**	**96.36**	**91.38**

**Table 6:** Ablation study results on the real-world transportation dataset (days).

Method	$F_{1}$ (%)	Precision (%)	Recall (%)
Informer baseline †	94.02	93.22	94.83
TCF-Trans w/o temporal context fusion †	85.22	85.96	84.48
TCF-Trans w/o feature fusion †	94.74	96.43	93.10
**TCF-Trans †**	**96.55**	**96.55**	**96.55**

**Table 7:** Comparison of the anomaly detection performance of the proposed method with different optimisers in relation to the real-world transportation dataset (days).

Optimiser	$F_{1}$ (%)	Precision (%)	Recall (%)
SGD †	88.50	90.91	86.21
Adam † *	**96.55**	**96.55**	**96.55**

* Current optimiser for the proposed method.

**Table 8:** Comparison of the anomaly detection performance of the proposed method with different ratios of training data on the real-world transportation dataset (days).

Ratio of Data (%)	$F_{1}$ (%)	Precision (%)	Recall (%)
80 †	92.86	96.30	89.66
90 †	94.74	96.43	93.10
100 † *	**96.55**	**96.55**	**96.55**

* Default ratio of data for the proposed method used in training.

**Table 9:** Comparison of the anomaly detection performance of the proposed method with different lengths of input and reference sequence on real-world transportation dataset (days).

Input and Reference Length	$F_{1}$ (%)	Precision (%)	Recall (%)
[3 & 1] †	93.91	94.74	93.10
[5 & 1] †	94.02	93.22	94.83
[5 & 2] †*	**96.55**	**96.55**	**96.55**
[7 & 2] †	93.81	96.36	91.38
[9 & 3] †	91.67	88.71	94.83

* Current lengths of input and reference sequence.

**Table 10:** Comparison of the anomaly detection performance of the proposed method with a four-layer decoder on the real-world transportation dataset (days).

Method	$F_{1}$ (%)	Precision (%)	Recall (%)
4-layer Informer baseline †	85.22	85.96	84.48
4-layer TCF-Trans †	93.91	94.74	93.10
**TCF-Trans †**	**96.55**	**96.55**	**96.55**

**Table 11:** Comparison of the anomaly detection performance of the proposed method with different types of training loss on the real-world transportation dataset (days).

Optimiser	$F_{1}$ (%)	Precision (%)	Recall (%)
MAE †	94.02	93.22	94.83
SmoothL1 †	95.73	94.92	**96.55**
MSE † *	**96.55**	**96.55**	**96.55**

* Current training loss for the proposed method.

## Data Availability

Not applicable.
